# Supplementary material for: Sexually dimorphic control of gene expression in sensory neurons regulates decision-making behavior in C. elegans
Source: eLife. 2017 Jan 24;6:e21166. doi: 10.7554/eLife.21166 (PMC5262377; doi:10.7554/eLife.21166)
Supplement: Supplementary file 1. — A comprehensive list of the strains used in this study. With the exception of CB1490, all strains are previously unpublished and were constructed for this study. DOI: http://dx.doi.org/10.7554/eLife.21166.015 [file elife-21166-supp1.docx]

**Supplementary File 1. *C. elegans* strains used in this study**

| **STRAIN** | **GENOTYPE** |
| --- | --- |
| CB1490 | *him-5(e1490)* |
| ZD1005 | *ksIs2[pdaf-7::GFP+rol-6(su1006)];him-5(e1490)* |
| ZD1157 | *daf-7(e1372); him-5(e1490)* |
| ZD1306 | *mgIs40;him-5(e1490); jxEx100[ptrx-1::ICE + pofm-1::GFP]* |
| ZD1307 | *mgIs40;him-5(e1490); jxEx102[ptrx-1::ICE + pofm-1::GFP]* |
| ZD1308 | *daf-7(e1372); him-5(e1490); daf-3(e1376)* |
| ZD1309 | *daf-7(ok3125); him-5(e1490)* |
| ZD1350 | *daf-7(ok3125); him-5(e1490); daf-3(e1376)* |
| ZD1386 | *daf-7(ok3125); him-5(e1490); qdEx37[pdaf-7::daf-7 + pges-1::GFP]* |
| ZD1388 | *daf-7(ok3125); him-5(e1490); qdEx44[pstr-3::daf-7 + pges-1::GFP]* |
| ZD1389 | *daf-7(ok3125); him-5(e1490); qdEx41[ptrx-1::daf-7 + pges-1::GFP]* |
| ZD1453 | *daf-1(m402); him-5(e1490)* |
| ZD1454 | *daf-1(e1287); him-5(e1490)* |
| ZD1482 | *him-5(e1490); ofEx4[pBLH98: lin-15(+) + trx-1::GFP]* |
| ZD1559 | *ksIs2; lov-1(sy582); pkd-2(sy606); him-5(e1490)* |
| ZD1573 | *ksIs2; mab-3(e1240); him-5(e1490)* |
| ZD1574 | *ksIs2; him-5(e1490); ceh-30(n4289)* |
| ZD1624 | *ksIs2; him-5(e1490); qdEx137[prab-3::tra-2IC::SL2::mCherry + pofm-1::GFP]* |
| ZD1625 | *ksIs2; him-5(e1490); qdEx138[prab-3::fem-3(+)::SL2::mCherry + pofm-1::GFP]* |
| ZD1628 | *ksIs2; dpy-18(e1096)/tra-1(e1099)* |
| ZD1723 | *ksIs2; him-5(e1490); qdEx139[ptrx-1::tra-2IC::SL2::mCherry+pofm-1::GFP]* |
| ZD1727 | *ksIs2; him-5(e1490); qdEx140[pbbs-1::tra-2IC::SL2::mCherry+pofm-1::GFP]* |
| ZD1765 | *ksIs2; him-5(e1490); qdEx144[ptrx-1::fem-3(+)::SL2::mCherry + pofm-1::GFP]* |
