# Supplementary material for: Sexually dimorphic control of gene expression in sensory neurons regulates decision-making behavior in C. elegans
Source: eLife. 2017 Jan 24;6:e21166. doi: 10.7554/eLife.21166 (PMC5262377; doi:10.7554/eLife.21166)
Supplement: Supplementary file 2. — A comprehensive list of the oligos used for constructing the transgenic strains created for this study. All sequences are listed in the 5’ to 3’ direction. DOI: http://dx.doi.org/10.7554/eLife.21166.016 [file elife-21166-supp2.docx]

**Supplementary File 2. Oligos used for transgenic strain generation**

All sequences are listed in the 5’ to 3’ direction.

| **Oligos used to generate neural masculinization construct** |  |
| --- | --- |
| ATGGTCTCAAAGGGTGAAG | pGH8 forward primer |
| TAAGCCTGCTTTTTTGTACAAAC | pGH8 reverse primer |
| agtagccctattttcagATGGAGGTGGATCCGGGT | fem-3 forward w/prab-3 overlap |
| gttgaagagtaattggacTCATCGTTTCCTGGAGCAATC | fem-3 reverse w/ unc-54 overlap |
| gaaacgatgaGTCCAATTACTCTTCAACATCC | unc-54 3' UTR forward w/fem-3 overlap |
| atgagacagcTTCAAAAAAATTTATCAGAAGTAAAAAAC | unc-54 3'UTR reverse w/SL2 overlap |
| atttttttgaaGCTGTCTCATCCTACTTTCAC | SL2 forward w/unc-54 overlap |
| tcttcaccctttgagaccatGATGCGTTGAAGCAGTTTC | SL2 reverse w/mCherry overlap |
| **Oligos used to generate neural feminization construct** |  |
| agccctattttcagatgGAATTCTCAATCAAACGATC | tra-2IC forward w/prab-3 overlap |
| gttgaagagtaattggacTTAAACCTCTGGGTCTGATAG | tra-2IC reverse w/unc-54 overlap |
| gaggtttaaGTCCAATTACTCTTCAACATCC | unc-54 forward w/tra-2IC overlap |
| agtaggatgagacagcTTCAAAAAAATTTATCAGAAGTAAAAAAC | unc-54 reverse w/SL2 overlap |
| **Oligos used for other feminization constructs** |  |
| ATGGAATTCTCAATCAAACG | Feminization linearization forward (no promoter) |
| CAACTTTTCTATACAAAGTTGATAGC | Feminization linearization reverse |
| actttgtatagaaaagttgTGAGTTGGCACTTCGTAG | trx-1p forward w/backbone overlap |
| gaattccatGATCAATTGCTCAAAGTCAC | trx-1p reverse w/tra-2IC overlap |
| actttgtatagaaaagttgTTGCAAAGAAGCACATCA | bbs-1p forward w/ backbone overlap |
| gtttgattgagaattccatTTTTTGTTAATTTTGGAGCAC | bbs-1p reverse w/ tra-2IC overlap |
| actttgtatagaaaagttgGGTCGAGCTGAATACACG | elt-2p Forward w/ backbone overlap |
| gtttgattgagaattccatTCTATAATCTATTTTCTAGTTTCTATTTTATTAG | elt-2p reverse w/tra-2IC overlap |
| **Oligos used for other masculinization constructs** |  |
| ATGGAGGTGGATCCGGGT | Masculinization linearization reverse (use with feminization linearization forward) |
| cggatccacctccatGATCAATTGCTCAAAGTCAC | trx-1p R w/fem-3 overlap (use with F listed above) |
